# A minimally invasive approach to induce myocardial infarction in mice without thoracotomy

**DOI:** 10.1111/jcmm.13708

**Published:** 2018-09-14

**Authors:** Quan Sun, Kang‐Kai Wang, Miao Pan, Ji‐Peng Zhou, Xue‐Ting Qiu, Zhen‐Yu Wang, Zhen Yang, Yan Chen, Hong Shen, Qi‐Lin Gu, Long‐Hou Fang, Guo‐Gang Zhang, Yong‐Ping Bai

**Affiliations:** ^1^ Department of Cardiovascular Medicine Xiangya Hospital Central South University Changsha China; ^2^ Department of Pathophysiology Xiangya School of Medicine Central South University Changsha China; ^3^ Department of Geriatric Medicine Xiangya Hospital Central South University Changsha China; ^4^ Department of Hypertension and Vascular Disease the First Affiliated Hospital Sun Yat‐Sen University Guangzhou China; ^5^ Department of Hematology Xiangya Hospital Central South University Changsha China; ^6^ Institute of Medical Sciences Xiangya Hospital Central South University Changsha China; ^7^ Department of Cardiovascular Sciences Houston Methodist Research Institute Houston TX USA

**Keywords:** cardiac dysfunction, ischaemia/reperfusion, mouse model, myocardial infarction

## Abstract

Acute myocardial infarction (MI) is a leading cause of morbidity and mortality in the world. Traditional method to induce MI by left coronary artery (LCA) ligation is typically performed by an invasive approach that requires ventilation and thoracotomy, causing serious injuries in animals undergoing this surgery. We attempted to develop a minimally invasive method (MIM) to induce MI in mice. Under the guide of ultrasound, LCA ligation was performed in mice without ventilation and chest‐opening. Compared to sham mice, MIM induced MI in mice as determined by triphenyltetrazolium chloride staining and Masson staining. Mice with MIM surgery revealed the reductions of LVEF, LVFS, E/A and ascending aorta (AAO) blood flow, and the elevations of S‐T segment and serum cTn‐I levels at 24 post‐operative hours. The effects of MI induced by MIM were comparable to the effects of MI produced by traditional method in mice. Importantly, MIM increased the survival rates and caused less inflammation after the surgery of LCA ligation, compared to the surgery of traditional method. Further, MIM induced angiogenesis and apoptosis in ischaemic hearts from mice at postoperative 28 days as similarly as traditional method did. Finally, the MIM model was able to develop into the myocardial ischaemia/reperfusion model by using a balloon catheter with minor modifications. The MI model is able to be efficiently induced by a minimally invasive approach in mice without ventilation and chest‐opening. This new model is potentially to be used in studying ischaemia‐related heart diseases.

## 1 INTRODUCTION

Ischaemic heart disease, caused by atherosclerosis, hypertension and vascular stiffness, etc, is a leading cause of death worldwide.^1^, ^2^, ^3^, ^4^ Acute myocardial infarction (MI) is the most common manifestation of ischaemic heart disease and represents a major cause of morbidity and mortality.^5^ A high‐efficient and less‐injured MI animal model is required to understand the pathophysiological processes and treatment strategies in basic medical studies, such as nitrate tolerance, vulnerable plaque, collateral artery formation and endothelial dysfunction.^6^, ^7^, ^8^, ^9^, ^10^, ^11^

Traditionally, a ventilation‐based thoracotomy to establish MI model by ligating left main descending coronary artery (LCA) in mice has been presented firstly by Johns and Olson in 1954, in which many surgical manipulations have been made to induce the cardiac ischaemic event.^12^ Although LCA ligation remains the most commonly practiced ischaemic injury, this remains to utilize methodology requiring ventilation and full opening of the chest, resulting in extensive tissue damages and high surgery‐related death.^13^ To solve these problems, Gao et al^14^, ^15^ improved a traditional method to induce MI injury in mice without ventilation. However, this method also needs to open the thoracic cavity rapidly because LCA is not visible if the chest is closed. Thoracotomy may cause some traumas, which not only increases animal pains and mortalities, but also exerts some detrimental effects on the whole body immune homoeostasis, a situation that limits clinical relevance to human patients.^16^

In this study, we established a new method to induce MI in mice without opening the chest and without ventilation. This minimally invasive method, (MIM) including identification of LCA, the transthoracic puncture and LCA ligation, was performed with the assistant of ultrasound. The new method was comparable to the traditional method to induce MI in mice, but it reduced the postoperative death rate and inflammatory response. Furthermore, we have developed this model to the new model of ischaemia/reperfusion (I/R) injury in mice, indicating the wide applications of this surgery.

## 2 MATERIALS AND METHODS

### 2.1 Reagents and animals

Primary antibodies against CD31 were obtained from Cell Signaling Company. All drug concentrations were expressed as the final molar concentration in the buffer. Male wild‐type (C57BL6) mice, 8‐12 weeks of age, 20‐25 g of body weight, were obtained from the Jackson Laboratory (Bar Harbor, ME). Mice were housed in temperature‐controlled cages with a 12‐hour light‐dark cycle and given free access to water and normal chows. All animal studies were conducted at the Animal Institute of Central South University according to the protocols approved by the Medical Experimental Animal Care Commission of Central South University.

### 2.2 Protocols of permanent coronary artery occlusion without opening thoracic cavity

1. Sterilize surgical instruments with a dry bead sterilizer (Germinator 500).
2. Mouse (generally 2‐3 months of age or at least 18 g body weight) is anaesthetized with 2%‐3% isoflurane inhalation in an inducing chamber.
3. Once anaesthetized, the mouse is removed from the inducing chamber to the surgical board, immobilized with tape and continuously anaesthetized with 2% isoflurane via coaxial breathing apparatus but not ventilated.
4. Remove the fur with a standard depilatory (eg, Nair) and clean the skin with water and then betadine and alcohol pads. To perform this procedure more efficiently, the step of fur‐removing could be done earlier.
5. Two small incisions (0.5 cm long) are made on the left and right chest skin with the scissors to expose the 3rd intercostal space as shown in Figure 1A.

**Figure 1:**
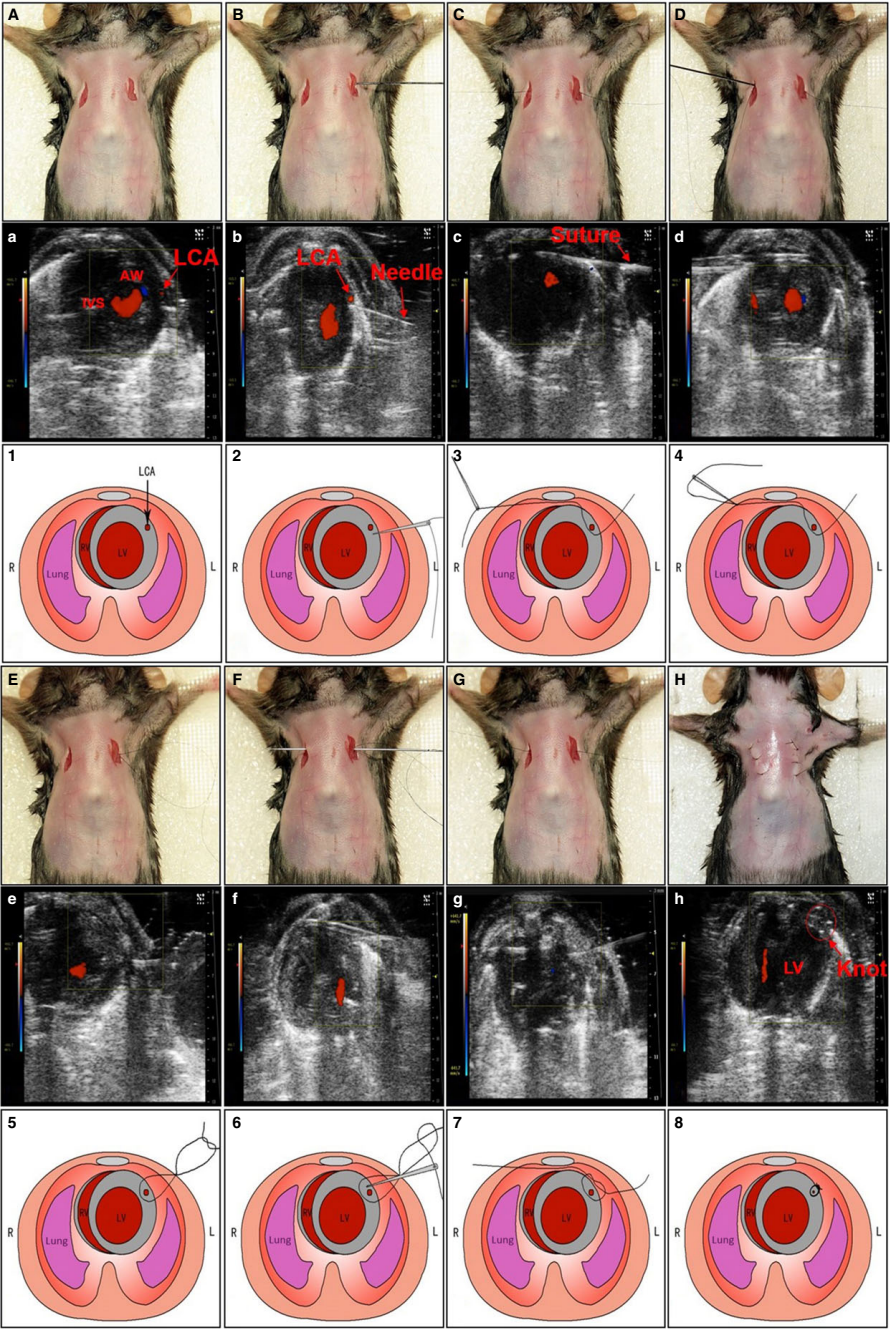
Permanent coronary artery occlusion without thoracotomy in mice under the guide of ultrasound. The detailed surgical procedures of coronary artery ligation in mice were described in the Section of Methods and presented in Video S1. A‐H, Images show the surgical procedures. (a‐h) Each operative stepwise in (A‐H) was monitored and recorded by ultrasound. (1‐8) The schematic presentations of these surgical procedures in (A‐H). Forty mice were used for practices
6. Echocardiography is performed using a VEVO 2100 imaging system (Visual Sonics Inc., Toronto, Canada) with a 30 MHz phased array transducer and a frame rate of 235/s. The echocardiography probe (MS‐400) is placed perpendicular to the sagittal plane of the chest within the 3rd intercostal space, imaging the left ventricle (LV) short axis. The LCA is shown in Figure 1a under ultrasound.
7. A small straight needle (0.2 mm in diameter) was inserted at the costal angle of the superior margin of the 3rd rib in the left chest. When it reaches the inferior of LCA (Figure 1B,b), we let the needle tip go up a little by gently pressing the side of needle attached by a 8‐0 silk suture. Under the guide of ultrasound, the heart surface is punctured by the straight needle and the needle is coming out of skin from the right chest (Figure 1C,c).
8. Then, the needle is inserted back from the right to of the left (Figure 1D). When the needle passes through the heart, it goes through above LCA (Figure 1E) under ultrasound and came out the skin from the same site in the left chest. For this step, the needle only passes through the space between the anterior chest wall and the anterior wall of the heart by gently pressing down both sides of suture to expand the space.
9. Once a loose knot is made (Figure 1F), the needle is inserted back from the left to the right in the chest. The LCA is now located inside of the knot (Figure 1G).
10. Ligation of LCA by pulling the two ends of the suture (Figure 1H) carefully. The knot is readily visible under ultrasound (Figure 1h). The ligation is proved by the typical ischaemic change in ECG (S‐T segment elevation). If S‐T elevation was not observed during the MIM surgery, the animal should be excluded.
11. The mouse is then allowed to breathe room air and monitored on a heating blanket during the recovery period, which is generally complete within 3‐5 minutes.
12. The sham group undergoes the same surgical procedures except that the LCA is not occluded.
13. One dose of buprenorphine (0.1 mg/kg) is administered subcutaneously and immediately after the incision is closed.
14. Clean surgical tools with PBS and alcohol.

For detailed procedures, please watch Video S1.

### 2.3 Traditional method to ligate LCA with ventilation and thoracotomy

The traditional, ventilation‐based method of MI in mice has been fully described by previous investigators with minor modifications.^17^

### 2.4 ECG analysis

Quantification of ECG was recorded before and after CICAL procedure using electrophysiological recording system (Taimeng Inc., Chengdu, China) as described previously.^18^ Otherwise, changes in ECG in echocardiography imaging system were also recorded during the whole CICAL and CICAL I/R procedures. Briefly, the electrodes of the plate were connected with three mouse limbs skin to monitor the changes in ECG in echocardiography.

### 2.5 Measurement of plasma cTn‐I levels

The mice were killed 24 hours after the surgery. ELISA kits were used to assess the levels of serum cTn‐I (Life Span Bio Sciences, Seattle, USA), following the manufacturer's instructions.

### 2.6 Microfil angiography

Microfil (Flow Tech Incorporation, MA, USA) was injected into the left ventricle until it accumulated in the coronary vessels. After the casting material was completely hardened, the perfused organ was removed and placed in paraformaldehyde for several hours. Images were acquired on a Leica M205 FC stereo microscope.

### 2.7 TTC staining

As described previously,^19^ the heart was rapidly excised 24 hours after surgical procedures and then washed for three times in cold PBS. Hearts were sliced into 1.0‐mm‐thick sections transversely from the apex alongside the long axis as shown inFigure  S1. Transverse sections were incubated in 1% TTC (Sigma Inc., USA) in a phosphate solution (pH 7.4) for 20 minutes at 37°C, and then transferred to 10% formalin, and incubated for 20 minutes at room temperature. Images were acquired by a regular camera.

### 2.8 Masson's trichrome staining‐based evaluation of myocardial injury

Mice were killed 24 hours and 4 weeks after the MI surgery. The heart was fixed with 4% paraformaldehyde overnight at room temperature, dehydrated sequentially through ethanol, butyl alcohol and embedded in paraffin. Longitudinal sections (4 μm) were stained with Masson's trichrome. Images were acquired on Panoramic MIDI (3D HISTECH Inc., Hungary), and the myocardial infarct size was calculated via Image‐pro plus software. Infarct size was determined as the percentage of epicardial and endocardial circumference occupied by the infarct on all heart slices.

### 2.9 Echocardiography measurements in vivo

After anaesthetization, individual mouse was placed on a heating pad (37°C) and underwent echocardiography using a VEVO 2100 micro‐ultrasound system with the echocardiography probe (MS‐400). Percentage changes in both LVEF and LVFS, the peak velocity (Vmax) of the AAO blood flow and E and A peaks were calculated and recorded using Visual Sonic analysis software (Visual Sonics Inc. Toronto, Canada).

### 2.10 Determination of angiogenesis by immunohistochemistry

Histological analysis was assessed in perfusion/fixed hearts collected from mice at 28 post‐operative days. The right atrium was then cut, and the myocardial vasculature was perfused, followed by 10 minutes perfusion with 10% formalin. The hearts were harvested and fixed in 4% formalin for 24 hours. The formalin‐fixed tissues were embedded in paraffin wax and cut into 4 μm sections. For the measurement of capillary density (counts/mm^2^), we performed immunohistochemical analysis of CD31 as described previously.^20^

### 2.11 Apoptosis assay

Tissues were fixed with 4% paraformaldehyde in PBS. Apoptosis was assessed by terminal deoxynucleotidyl transferase‐mediated dUTP nick‐end labelling (TUNEL) staining (tetramethylrhodamine red) using a kit from Roche Applied Science [Indianapolis, IN, USA])and following the provided instruction manual. The percentage of apoptosis was calculated from the number of TUNEL‐positive cells divided by the total number of cells counted as described previously.^21^

### 2.12 Statistical analysis

All quantitative results are expressed as mean ± SD. Statistical comparisons between two groups were analysed by unpaired Student's *t* test. Multiple comparisons were analysed with a one‐way ANOVA followed by Tukey post hoc tests or Bonferroni post hoc analyses. The comparisons of the Kaplan‐Meier survival curves were analysed with Log Rank statistical method. A two‐sided *P*‐value <.05 was considered significant.

## 3 RESULTS

### 3.1 Novel MIM is successful to establish MI model in mice

As described in Section of Methods, we used the new method to induce MI injury without ventilation and thoracotomy. To determine whether this new method is efficiently to induce MI, we visualized the heart and coronary arteries using microfil angiography and examined the MI size as described previously.^22^ In all cohort (63 mice) used for MIM surgery, 2 mice died of LCA angiorrhexis within 6 hours after the surgery, and 3 mice were excluded because S‐T enhancement was not observed during the MIM surgery. We did not observe the major complications such as pneumothorax in all mice.

**Figure 2:**
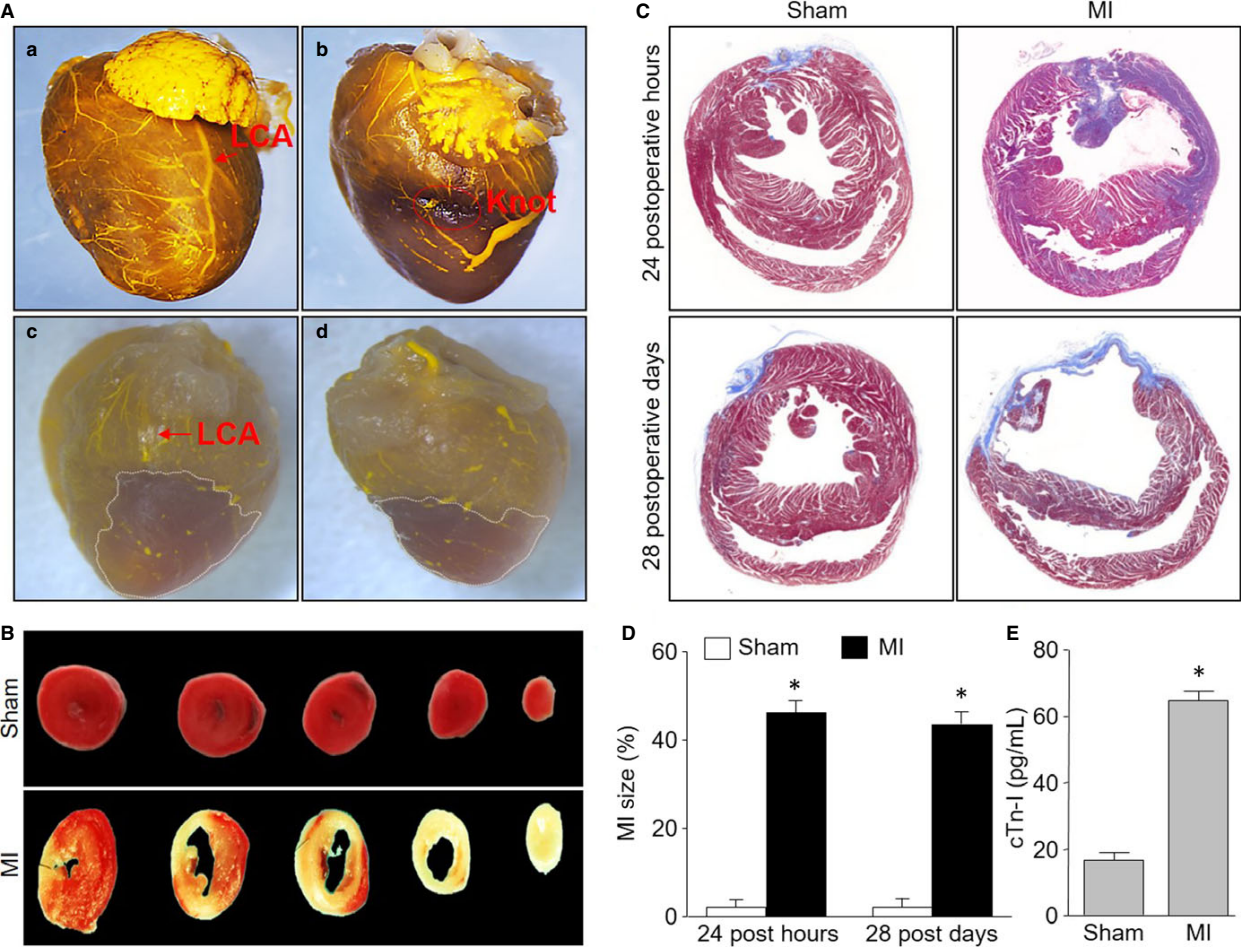
Evaluations of myocardial infarction (MI) in mice with the surgery of ultrasound‐based left coronary artery (LCA) ligation. The MI was induced by LCA ligation as described in Figure 1 and Section of Methods. A, Heart vessels were visualized by using microfil in mice. Images of microfil angiography showing heart vessels in sham mice (a) and LCA‐ligated mice (b). The areas of myocardial infarction from LCA‐ligated mice were outlined by dotted lines from the anterior (c) and the posterior (d). B, TTC staining at 24 post‐operative hours after minimally invasive method surgery or sham. C, Masson's trichrome‐staining was performed at hearts from mice at 24 post‐operative hours and 28 post‐operative days after LCA ligation or sham. D, Quantitative analysis of the MI size in pictures from (C). E, Serum cTn‐I level was determined at 24 post‐operative hours in mice. *P <* *.05 vs Sham. N = 15‐20 per group. Unpaired Student's *t* test was used for statistical comparisons in (D,E)

As shown in Figure 2A‐a,b, microfil angiography clearly outlined the LCA, which was ligated in mice hearts with the MIM surgery. The area of MI was also evidently identified by the microfil angiography imaging (Figure 2A‐c,d). The area of MI was also examined by TTC staining (Figure 2B) and Masson's trichrome‐staining (Figure 2C). Quantitative analysis of Masson's trichrome‐staining indicated that the new method of MIM caused significant heart ischaemia in mice (Figure 2D). These results demonstrate that the novel MIM successfully induced MI model in mice without ventilation and thoracotomy.

### 3.2 Serum cTn‐I level is increased in mice after the MIM surgery

Acute MI, defined as an elevation of cardiac troponin I (cTn‐I) resulting from ischaemia, is associated with substantial mortality in surgical patients.^23^ Thus, we assayed serum cTn‐I levels to further confirm the effects of MI induced by MIM. As indicated in Figure 2E, serum cTn‐I levels were totally increased at 24 postoperative hours, compared to sham mice, suggesting the new method is effective to induce myocardial injury in mice.

### 3.3 Cardiac dysfunction is induced by new method in mice

**Figure 3:**
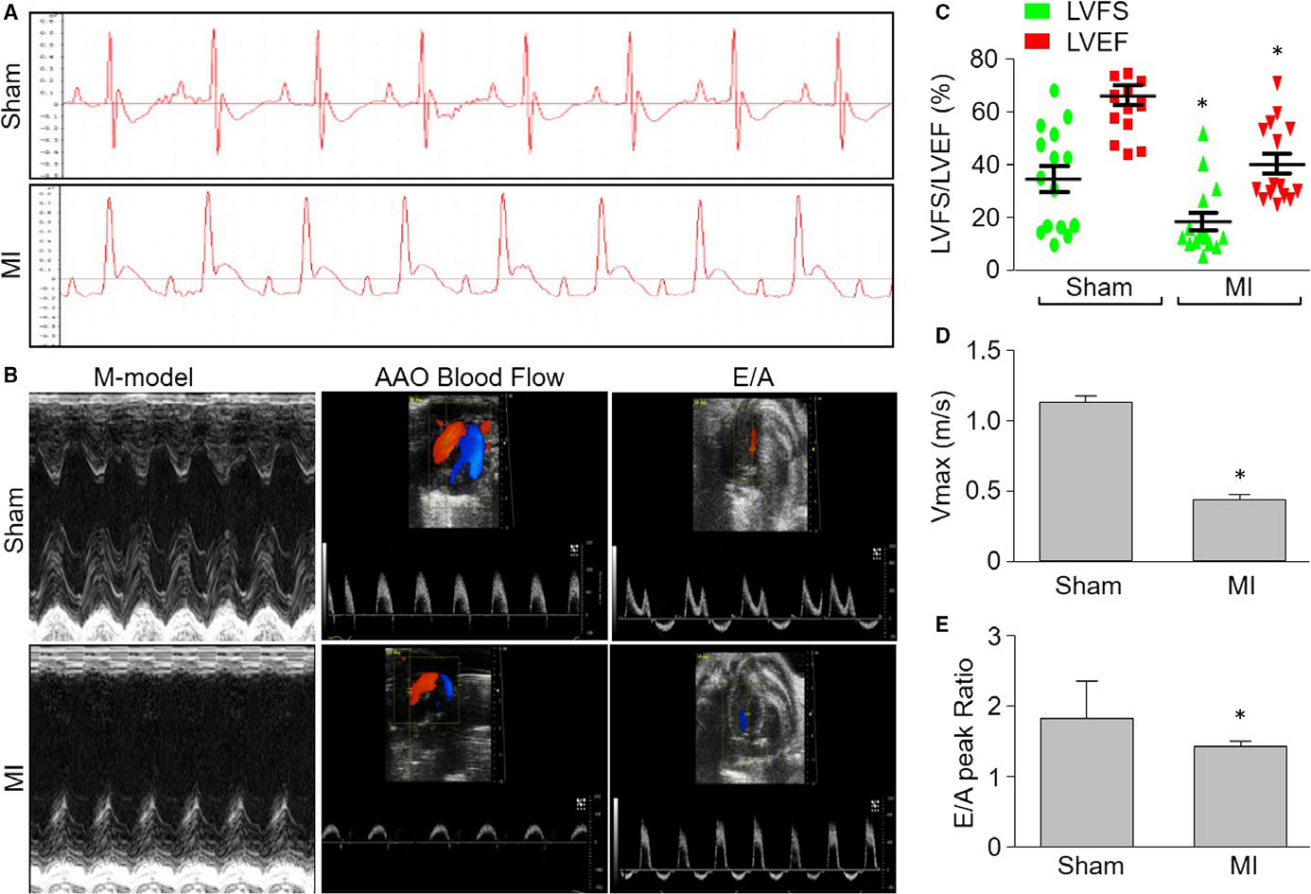
Cardiac dysfunction induced by minimally invasive method (MIM) surgery without opening thoracic cavity in mice under the guide of ultrasound. Myocardial infarction was induced by MIM‐based left coronary artery (LCA) ligation as described in Figure 1 and Section of Methods. A, ECG measurements were performed using the electrophysiological recording system during the surgery. B, Representative echocardiographic images showing the kinetics of anterior wall motion, ascending aorta (AAO) blood flow and the E/A peaks in mice 28 d after LCA ligation. C, Quantitative analyses of LVEF and LVFS in pictures from (B). D, Quantitative analysis of AAO blood flow in pictures from (B). E, Quantitative analysis of the E/A peak ratio in pictures from (B). *P <* *.05 vs Sham. N = 15‐20 per group. Unpaired Student's *t* test was used for statistical comparisons in (C,D,E)

Cardiac dysfunction is a key factor contributing to the mortality in patients with MI.^24^ We then examined heart function to assess the effects of MI in mice with MIM surgery by electrocardiogram (ECG) at 24 post‐operative hours. As shown in Figure 3A, the S‐T segments were markedly elevated in mice with MIM surgery, compared to mice with sham surgery. Moreover, both LVEF and LVFS were decreased in mice with MIM (Figure 3B,C), indicating the impaired anterior wall motion of the heart caused by the new method.

The systolic and diastolic functions of hearts were also determined by calculating the Vmax of ascending aorta (AAO) blood flow and the E/A peak ratio. As depicted in Figure 3B,D,E, both Vmax of AAO and the E/A peak ratio were noticeably decreased in mice with MIM surgery, compared with the mice following sham surgery, demonstrating that this new method is a useful approach to induce cardiac dysfunction in mice.

### 3.4 New method is comparable to traditional method to induce MI and cardiac dysfunction

**Figure 4:**
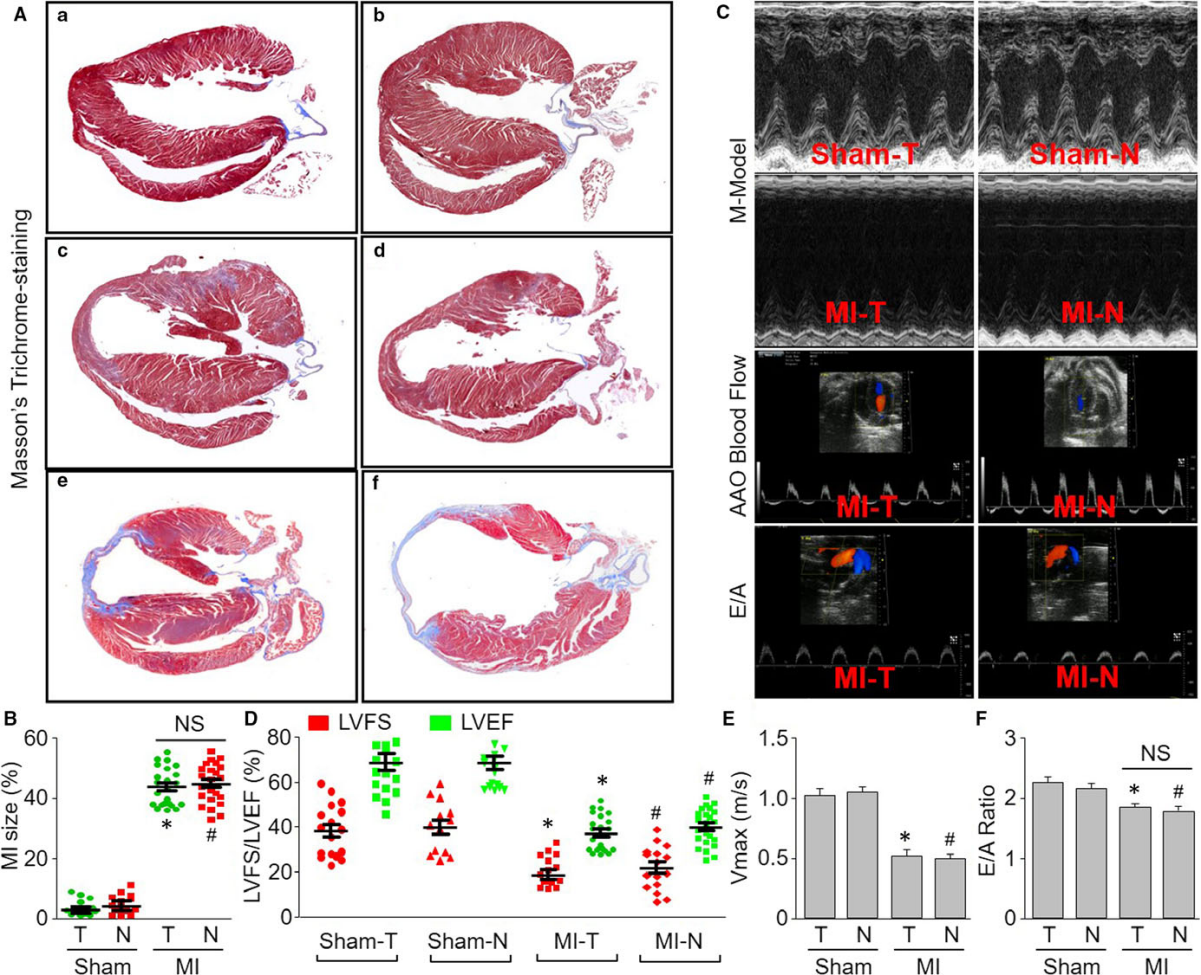
Assessments of the ischaemic effects induced by traditional method and new method in mice. The new method and traditional method to induce myocardial infarction (MI) were described in Section of Methods. A, Masson's trichrome‐staining was performed at heart from mice. (a) sham of traditional method (Sham‐T) at post‐operative 24 h. (b) sham of new method (Sham‐N) at post‐operative 24 h. (c) MI by traditional method (MI‐T) at post‐operative 24 h. (d) MI by new method (MI‐N) at post‐operative 24 h. (e) MI‐T at post‐operative 28 d. (f) MI‐N at post‐operative 28 d. B, Quantitative analysis of the MI size from (A). C, Representative echocardiographic images showing the kinetics of anterior wall motion, ascending aorta (AAO) blood flow and the E/A peaks in mice 28 d after left coronary artery ligation. D, Quantitative analyses of LVEF and LVFS in pictures from (C). E, Quantitative analysis of AAO blood flow Vmax in pictures from (C). F, Quantitative analysis of the E/A peaks in pictures from (C). *P <* *.05 vs Sham‐T. ^#^ *P *<* *.05 vs Sham‐N. NS indicates no significance. N = 15‐20 per group. One‐way ANOVA followed by Tukey post hoc tests was used in (B,D,E,F) for statistical analysis

We next assessed the ischaemic effects induced by traditional method and new method in mice. Masson's trichrome‐staining indicated that there were no any differences between new method and traditional method in MI mice (Figure 4A,B). Cardiac dysfunction, including LVEF/LVFS, the Vmax of AAO blood flow and the E/A peak ratio, was identical in both mice with MIM surgery and mice with traditional method surgery (Figure 4C‐F). These data demonstrate the new method is comparable to traditional method to induce MI and cardiac dysfunction in mice.

### 3.5 MIM surgery shortens recovery time and causes less inflammation

**Figure 5:**
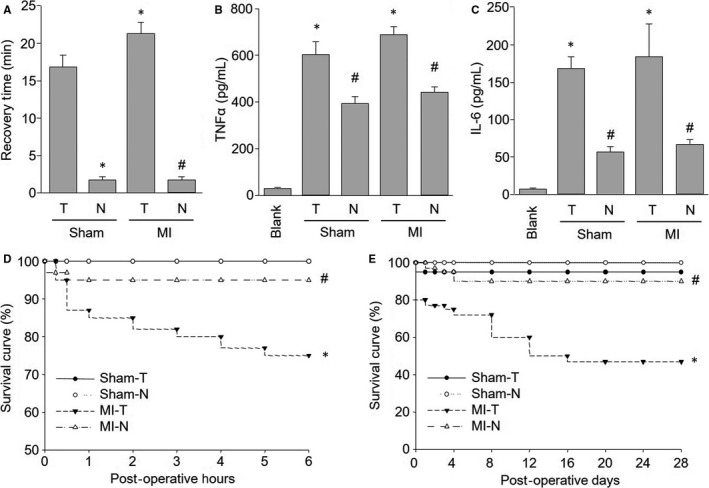
Comparisons of injuries caused by new method and traditional method in mice. The new method and traditional method to induce myocardial infarction in mice were described in Section of Methods. A, Recovery time in mice undergoing surgery. B,C, Cytokines including TNFα (B) and IL‐6 (C) in serum were assayed by ELISA in mice at post‐operative 28 d. D,E, The survival rates of short term (0‐6 h after surgery) in (D) and long term (0‐28 d after surgery) in (E) were calculated in mice. T, traditional method. N, new method. *P <* *.05 vs Sham‐T. ^#^ *P *<* *.05 vs MI‐T. N = 15‐20 per group. One‐way ANOVA followed by Tukey post hoc tests was used in (A,B,C) for statistical analysis. In (D,E), Kaplan‐Meier survival curves were compared using log rank statistical method

To evaluate the side injuries induced by MIM surgery, we compared the post‐operative recovery time in mice between undergoing new method and traditional method. The recovery time was defined as the interval from surgical completion to the recovery of animal consciousness or motion.^15^ As indicated in Figure 5A, the recovery time was significantly shortened in mice with sham surgery of new method than mice with sham surgery of traditional method. Further, the recovery time in mice with traditional method of MI surgery was longer than mice with sham surgery of traditional method, while there were no differences in mice between new method of sham surgery and new method with MI surgery, indicating LCA ligation in MIM surgery did not cause excessive injuries in mice.

To investigate the inflammatory response caused by new method, the plasma levels of TNFα and IL‐6 were measured at 24 hours after surgery. As shown in Figure 5B,C, both traditional method and new method significantly increased the plasma levels of TNFα and IL‐6, compared with mice without any surgery. Compared to sham mice, traditional method of MI surgery further increased the plasma levels of TNFα and IL‐6, while new method did not. Overall, the above data indicate that the new procedure causes less tissue damage and provides for faster recovery after surgery.

### 3.6 New method of MIM surgery increases survivals of mice

The short/long survival rate was also calculated in mice with new method and traditional method. As shown in Figure 5D, from the beginning of the surgery to 6 hours after surgery, no mice died when each sham surgery was performed. The survival rate in mice with MIM surgery was approximately 95%, while it was about 75% in mice with MI by traditional method. The overall survival rates (including surgery‐related death) 28 days after surgery was 100% in mice with sham surgery of new method and 95% in mice with sham surgery of traditional method (Figure 5E). The overall survival rate at the 28th post‐operative day was 90% in mice with MIM surgery, which was significantly higher than 45% in mice with MI by traditional method (Figure 5E). These data confirm an improved outcome using this new method of MI.

### 3.7 New method of MIM surgery induces angiogenesis

**Figure 6:**
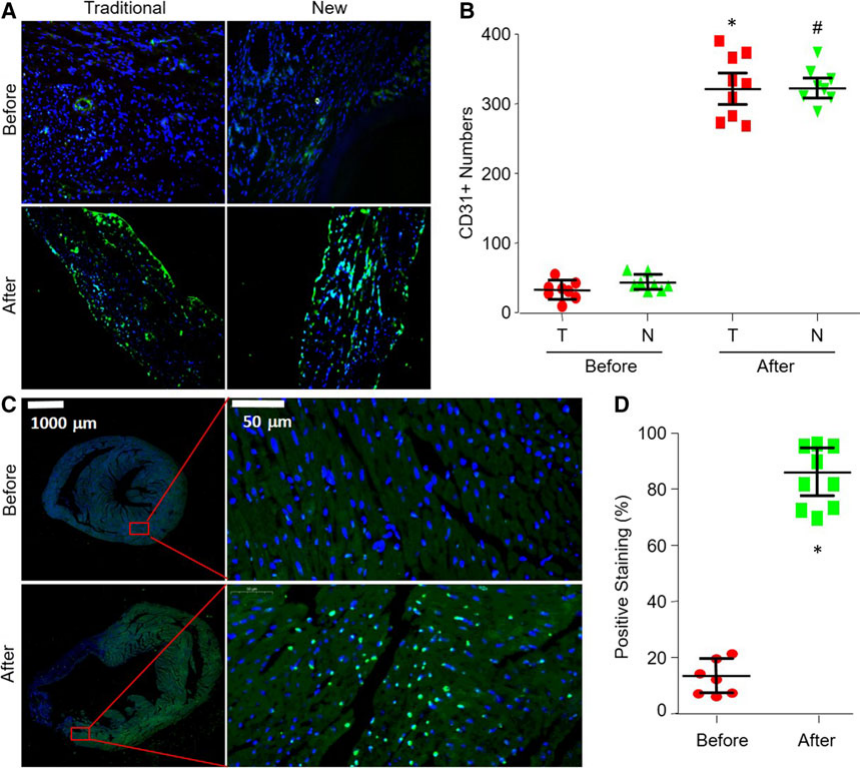
Applications of the new myocardial infarction (MI) model to study angiogenesis and apoptosis in mice. The new method and traditional method to induce MI were described in Section of Methods. A, Angiogenesis in ischaemic heart was determined by IHC analysis of CD31 in mice before the surgery of LCA ligation or at post‐operative 28 d undergoing LCA ligation through new method and traditional method. B, The quantitative analysis of CD31 was shown. T, traditional method. N, new method. *P <* *.05 vs before surgery of traditional method. ^#^ *P *<* *.05 vs before surgery of new method. C, Cell apoptosis in ischaemic heart was determined by TUNEL at post‐operative 28 d in mice before the surgery of LCA ligation or at post‐operative 28 d undergoing LCA ligation through new method. D, The quantitative analysis of TUNEL‐positive staining was shown. *P <* *.05 vs before surgery of new method. N = 5‐10 per group. One‐way ANOVA followed by Tukey post hoc tests was used in (B) for statistical analysis. Unpaired Student's *t* test was used for statistical comparisons in (D)

Angiogenesis is critical for re‐establishing the blood supply to the surviving myocardium after MI and, consequently, to the recovery of cardiac function.^25^ To test whether this model is suitable for studying angiogenesis, we detected the angiogenesis by analysis of CD31, which is a biomarker for endothelial cells.^26^ As shown in Figure 6A, ischaemia dramatically induced angiogenesis in mice with MI caused by both traditional method and new method, suggesting this new method is suitable for studying in ischaemia‐induced angiogenesis.

### 3.8 New method of MIM surgery induces cardiac myocyte apoptosis

Studies have also demonstrated an important role of apoptosis in ischaemic heart disease, contributing to myocyte cell death in MI and left ventricular remodelling.^27^ To examine whether this model is applicable to study cardiac myocyte apoptosis induced by MI, we detected the apoptosis by TUNEL. As shown in Figure 6B, the apoptosis rate of cardiac myocyte in ischaemic area was completely increased in mice with the surgeries of both traditional method and new method, compared to sham mice. It reveals that MIM surgery is the same effective as the traditional method to be used in studying ischaemia‐induced angiogenesis.

### 3.9 Application of MI model established by new method to I/R model

**Figure 7:**
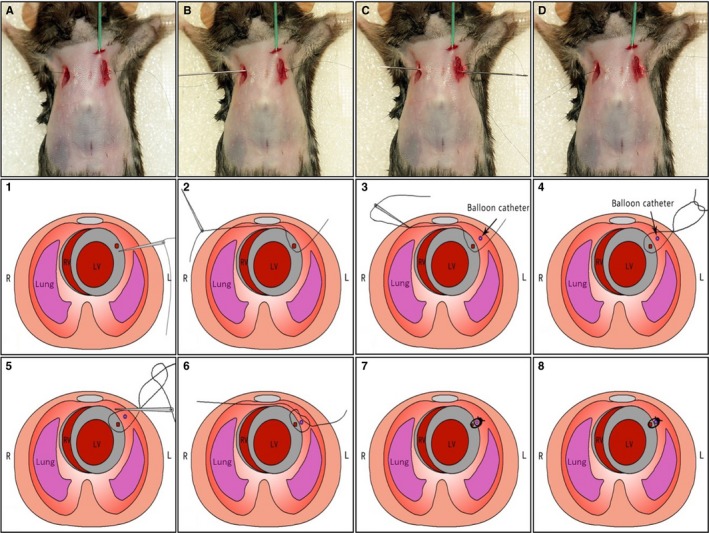
Development of permanent coronary artery occlusion without opening thoracic cavity to establish ischaemia/reperfusion (I/R) model in mice. This I/R injury procedure in mice is essentially the same as the procedure for inducing myocardial infarction as shown in Figure 1 except that a balloon catheter (1.25 F. Terumo, Hangzhou, China) is implanted through the first intercostal space into the thoracic cavity (parallel to the anterior median line) between anterior walls of chest and LV prior to make a tie (A‐D). The balloon is enclosed within the knot. Ischaemia and reperfusion were induced by inflating (lasting 30 min) and deflating (lasting 3 h) the balloon. Then, the balloon catheter was gently removed out of the chest (Step 1‐Step 8). All animals should be monitored after the surgery and should be given one dose (0.1 mg/kg) of buprenorphine within 6 h post‐surgery with another dose administered the following morning. No further analgesia is needed after that

**Figure 8:**
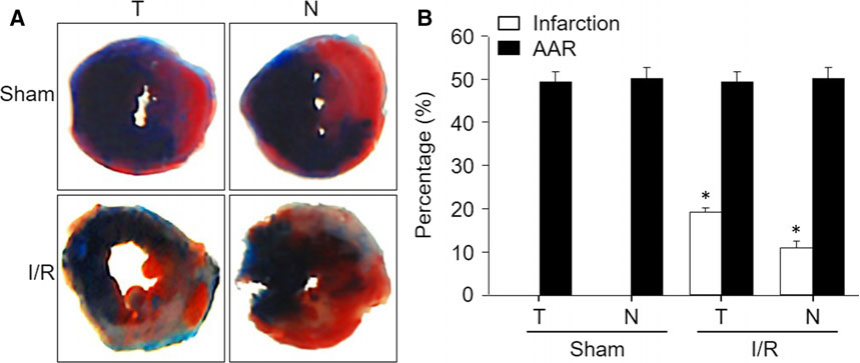
Comparisons of myocardial infarction (MI) size induced by MIM‐based ischaemia/reperfusion (I/R) and traditional I/R in mice. The new method and traditional method to induce I/R were described in Section of Methods. A, The AAR of ischaemia and the infarct area of the LV, as indicated by Evans blue plus TTC staining. B, Quantitative analysis of the MI size and AAR from (A). *P <* *.05 vs Sham. N = 5‐10 in each group. One‐way ANOVA followed by Tukey post hoc tests was used in (B) for statistical analysis

The I/R model is generally used to examine the short‐term consequences of ischaemic injury and has been used to investigate the development of innovative cardioprotective therapies.^14^ We finally established the I/R model based on this new method with some modifications. As shown in Figure 7, we placed a balloon catheter enclosed within the knot prior to LCA ligation. By inflating the balloon for 45 minutes and sequence deflating the balloon for 3 hours, we gently removed the balloon catheter out of the chest. The AAR of ischaemia and the infarct area of the LV, as indicated by Evans blue plus TTC staining, suggested that MIM surgery was successfully used to generate an I/R model in mice (Figure 8A). Moreover, the MI sizes were identical between the MIM‐based I/R group and the traditional I/R model group (28.09 ± 1.81% vs 27.64 ± 1.50%, Figure 8B). These results show that the modified MIM surgery is applicable in studying I/R injury in mice.

## 4 DISCUSSION

In this study, we described a minimally invasive approach to ligate coronary without thoracotomy under the guide of ultrasound. This new method is more efficient to induce MI and causes less injuries, compared to the traditional method in mice. Further, this method is suitable to be used in studying ischaemia‐induced angiogenesis and apoptosis.

The biggest advantage of this new MI model is that we do not open the chest and do not use any equipment to assist respiration. Traditional procedure to ligate LCA in mice is performed with opening the chest and with ventilation, which requires intubation and thoracotomies.^28^ Ren et al^29^ have shown that remote surgical trauma induces preconditioning of the heart. Other endogenous ligands such as high mobility group box 1 or heat‐shock proteins which are released or secreted following trauma have been shown to modulate myocardial function.^30^, ^31^, ^32^ Thus, tissue damages caused by tracheal intubation and chest‐opening not only threaten animal life, but also affect the accuracy of observations on the traditional MI model.^33^, ^34^ Gao et al^14^, ^15^ have developed a rapid surgical method to ligate LCA without ventilation, which improved the short‐term and long‐term survival rates in mice. However, this modified method is still conducted under the opened chest, causing huge pain and high death rate in mice. Both methods conflict with the 3‐R principles including Reduction, Replacement and Refinement of experimental animals.^35^ Although Kim et al^36^ claimed that they established a murine closed‐chest model of myocardial I/R using hanging weights, the thoracotomy was also performed during the surgical procedures. In the present study, we solidly generated a novel MI model in mice using ultrasound‐guided transthoracic puncture and closed intrathoracic LCA ligation. This would alleviate the surgery‐caused injury as possible.

A limitation of this new MI model is that we have to perform all surgical procedures under the guide of ultrasound. This depends not only on the in vivo imaging system with high‐resolution, but also on the excellent skills to identify LCA and perform heart puncture under ultrasound. The equipment of ultrasound is too expensive to be used generally. High technical challenge of heart puncture requires great efforts for unexperienced technicians. To master this technique, we have done a lot of practices on 50 mice to ensure the accuracy of LCA ligation. The advantage of skin incision combined with echocardiography may prevent accidental lung injury as the edge of lung is visible by echocardiography imaging system. Further, we may stop the puncture once the incorrect puncture of the heart was seen on echocardiography imaging system. Once this technique is mastered, the major complications such as pneumothorax, incorrect puncture of the heart or false targeted ligations were few. Anyway, good expertise on operating ultrasounds and doing puncture is essential to perform the MIM surgery.

A difficulty of this technique is that the real‐time image of the heart as viewed on the monitor will be distorted which is also reflected in the video, due to movement in the chest wall musculature as well as the LV wall. The same would happen every time when the needle is inserted and there is movement in musculature. Therefore, the ultrasound probe will have to be constantly re‐adjusted to get a real‐time accurate image of the heart, hence adding to the time taken to perform the procedure.

Another difficulty of this technique is how to control the depth of the needle so that it can precisely cross the LCA rather than punctures into the heart chamber. For this question, we should identify the LCA, needle and heart wall under ultrasound correctly (Figure 1b). When the needle reaches the inferior of LCA as monitored by ultrasound, the needle tip is going up a little by pressing the side of needle connected to the suture (Figure S2 as a flash and Video S2). This manipulation is critical to avoid the incorrect puncture of heart chamber. For this step, it is better to use a straight needle but not a curved needle to do puncture.

Frankly, the traditional method can be performed with a very small incision in the chest cavity and minimal trauma. The careful intubation and a weight based artificial respiratory equipment are mandatory to obtain safe ventilation during the procedure. Due to evacuation of the thorax, intensive care after the surgery and good pain management, the mortality rate of traditional operated LCA ligation can be reduced to a minimum. Furthermore, the correct ligation can be evaluated directly while performing the surgery of traditional method of LCA ligation. Also, the traditional method can be trained with dead and intubated animals while the MIM method has to be performed under the ultrasound of a living animal. For these reasons, the traditional method still has its advantages, compared to the MIM method.

In conclusion, we have efficiently established a minimally invasive MI model without thoracotomy and ventilation. This new model is not only applied to study ischaemia‐induced angiogenesis and apoptosis, but also developed to I/R model with minor modifications.

## CONFLICT OF INTEREST

The authors confirm that there are no conflict of interests.

## AUTHOR CONTRIBUTION

Q.S. designed and performed most experiments. K.K.W., M.P., J.P.Z., X.T.Q., Z.Y.W., Z.Y., Y.C., H.S., Q.L.G. and L.H.F. partially performed some experiments. G.G.Z. and Y.P.B. convinced the project, performed statistical analysis and wrote the manuscript. We thank Professor Shuang‐Xi Wang for his critical suggestions and excellent language editing.
