# Supplementary material for: A minimally invasive approach to induce myocardial infarction in mice without thoracotomy
Source: J Cell Mol Med. 2018 Sep 14;22(11):5208–19. doi: 10.1111/jcmm.13708 (PMC6201221; doi:10.1111/jcmm.13708)
Supplement: Supplementary file 1 [file JCMM-22-5208-s001.docx]

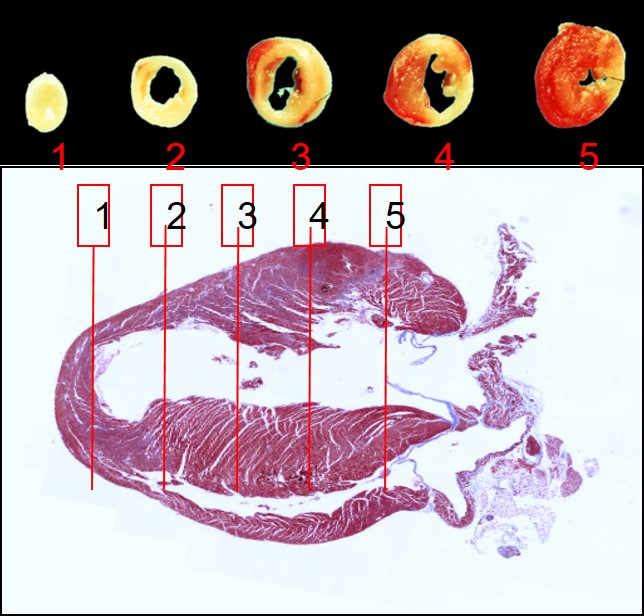


**Supplemental Figure 1**. How to get transverse sections from heart for TTC staining.


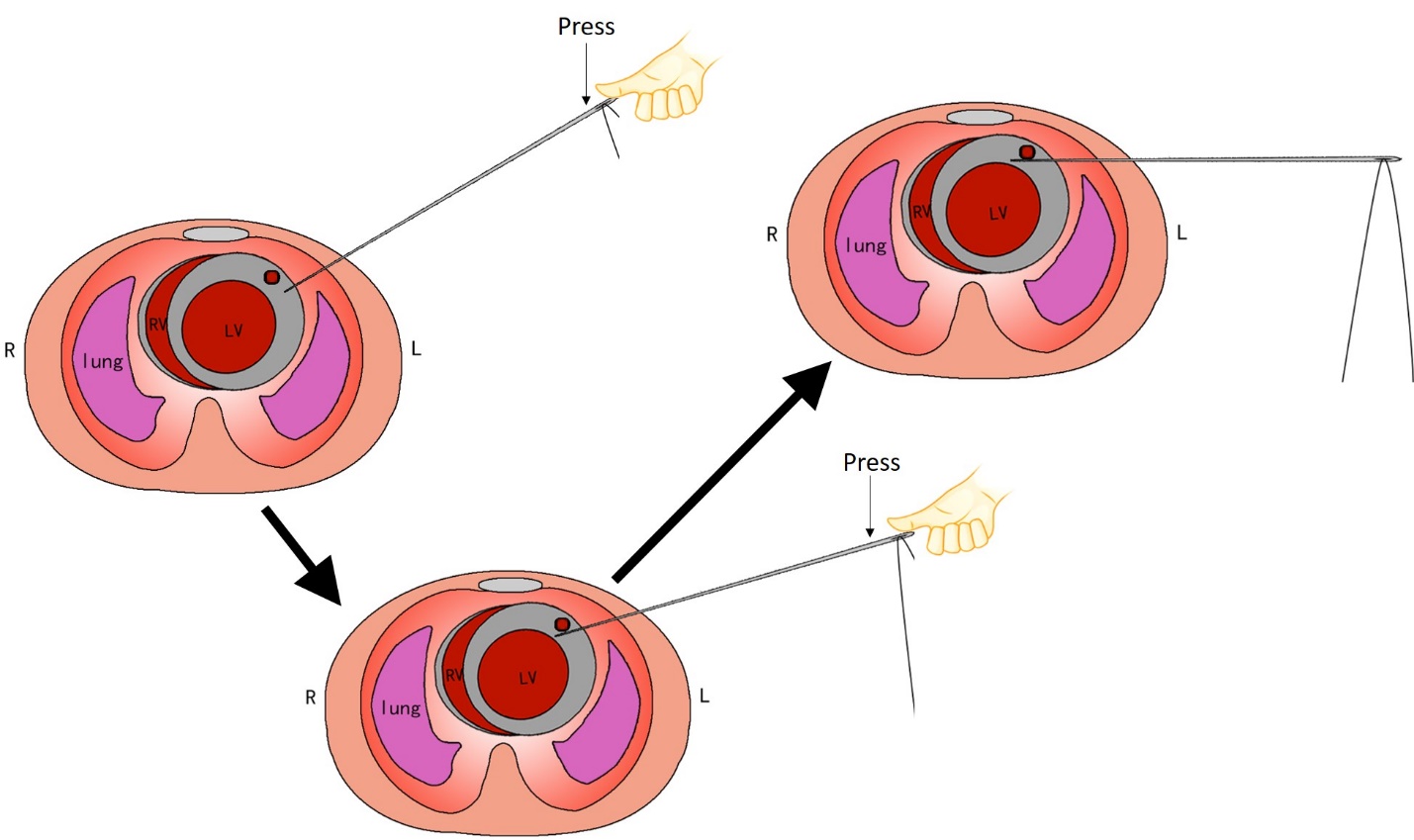


**Supplemental Figure 2**. The schematic presentations of the critical steps how to avoid puncturing into the heart chamber. When the straight needle reaches the inferior of LCA as monitored by ultrasound, we let the needle tip go up a little by gently pressing the side of needle connected to the suture.
